# Gyrase and Topoisomerase IV as Antibacterial Targets for Gepotidacin and Zoliflodacin: Teaching Old Enzymes New Tricks

**DOI:** 10.3390/ijms27010496

**Published:** 2026-01-03

**Authors:** Neil Osheroff

**Affiliations:** 1 Department of Biochemistry, Vanderbilt University School of Medicine, Nashville, TN 37232, USA; neil.osheroff@vanderbilt.edu; 2 Department of Medicine (Hematology/Oncology), Vanderbilt University School of Medicine, Nashville, TN 37232, USA

**Keywords:** gyrase, topoisomerase IV, antibacterial, fluoroquinolone, gepotidacin, zoliflodacin

## Abstract

The essential bacterial type II topoisomerases gyrase and topoisomerase IV have been exploited as the therapeutic targets of fluoroquinolone antibacterials for over four decades. Despite their broad utility, the effectiveness of fluoroquinolones has been increasingly undermined by the widespread emergence of target-mediated resistance, highlighting the need for alternative therapeutic strategies. Recent advances have produced two mechanistically distinct classes of gyrase/topoisomerase IV-targeted antibacterials: the triazaacenaphthylenes and the spiropyrimidinetriones. The first-in-class agents gepotidacin and zoliflodacin, respectively, were approved for human use in 2025, representing the first new antibacterial classes targeting these enzymes in decades. This commentary examines the mechanisms of action of these agents, contrasts their interactions with gyrase and topoisomerase IV relative to fluoroquinolones, and considers their potential to address resistance while preserving the long-term clinical viability of therapy directed against the bacterial type II topoisomerases.

## 1. Introduction

The bacterial type II topoisomerases gyrase and topoisomerase IV play critical roles in regulating the topological state of the genome [1,2,3,4,5,6]. Typically, gyrase relieves positive supercoiling ahead of replication forks and transcription complexes, while topoisomerase IV resolves knots and tangles in the bacterial genome and unlinks daughter chromosomes following replication [1,2,3,4,5,6]. These enzymes regulate DNA topology by introducing a transient break in one DNA duplex and transporting a second duplex through the DNA gate. During the DNA cleavage step of their catalytic cycles, gyrase and topoisomerase IV maintain genomic integrity by forming covalent bonds between the newly generated 5′-DNA termini and active site tyrosine residues [1,2,3,6,7]. This covalent enzyme-cleaved DNA complex has been termed the “cleavage complex” [1,2,3,6,7].

The cleavage complex is normally fleeting and is tolerated by the bacterial cell [1,2,3,6,7]. However, circumstances that stabilize the cleavage complex and prolong the presence of the covalent enzyme-cleaved DNA bond have two potentially lethal effects on bacteria: they rob the cell of the essential functions of gyrase and topoisomerase IV, and they lead to DNA strand breaks that can no longer be ligated by the enzymes and lead to fragmentation of the genome [2,4,7,8,9]. Consequently, the cleavage complexes of gyrase and topoisomerase IV, while critical to enzyme action, have become significant targets for antibacterial drug discovery.

## 2. Fluoroquinolones

**Figure 1:**
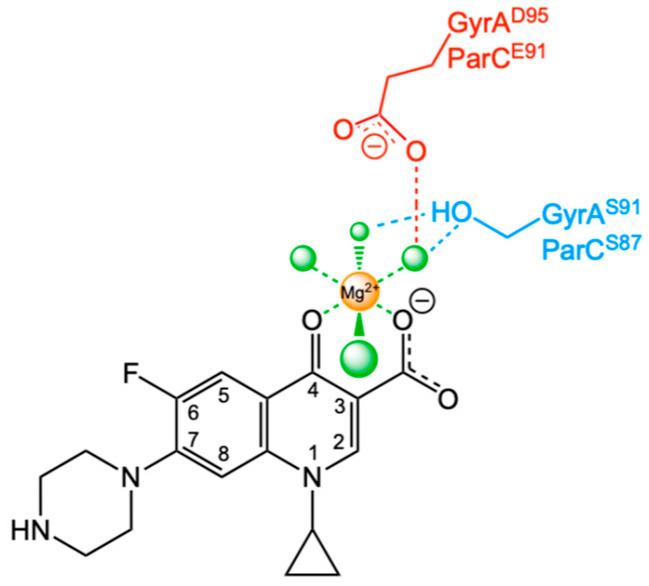
Representation of the water–metal ion bridge that mediates interactions between fluoroquinolones (ciprofloxacin) and bacterial type II topoisomerases. The bridge is formed between a non-catalytic divalent metal ion (orange, Mg^2+^), which is chelated by the C3/C4 keto-acid of cipro-floxacin (black). The metal ion is coordinated (green dashed lines) by four water molecules (green). Two of the water molecules form hydrogen bonds (blue dashed lines) with the serine side-chain hydroxyl (blue, *E. coli* GyrA^S83^/ParCS^80^), and one water molecule hydrogen bonds (red dashed lines) with the carboxylate side chain of aspartic acid or glutamic acid (red, *E. coli* GyrA^D87^/PArC^E84^).

Beyond their important cellular roles, gyrase and topoisomerase IV have for the past four decades made vital contributions to human health as the targets for the fluoroquinolone antibacterials [2,4,6,7,10,11,12,13]. Members of this drug class, including ciprofloxacin (Figure 1), moxifloxacin, and levofloxacin [12], are among the most widely prescribed antibacterials worldwide [12,14,15,16,17]. Fluoroquinolones were first approved for clinical use in the mid-1980s [12,14,15,16,17]. Since that time, they have been used to treat a broad spectrum of clinically important infections. Although fluoroquinolones have been designated by the World Health Organization as among the five “highest priority” critically important antimicrobial classes [16], their extensive use has been accompanied by the emergence of target-mediated resistance. This resistance is driven by specific mutations in gyrase and topoisomerase IV and has curtailed the clinical utility of this drug class [2,4,6,7,15].

Fluoroquinolones used in the clinic interact with the bacterial type II topoisomerases through a “water–metal ion bridge” (Figure 1). The bridge was first observed in the crystal structure of a cleavage complex formed by *Acinetobacter baumannii* topoisomerase IV and stabilized by moxifloxacin [18]. The water–metal ion bridge is formed through a divalent metal ion, which is chelated to the fluoroquinolone through its C3-C4 ketoacid and coordinated by four water molecules. The bridge is anchored to the protein through a serine residue (originally identified as serine 83 in the GyrA subunit of *Escherichia coli* gyrase) [19,20], which coordinates two of the water molecules and an acidic residue (aspartic or glutamic acid) four amino acids away (Glu87 in the *E. coli* GyrA subunit) [21], which coordinates one of the water molecules.

Although it had been known for decades that mutations in the above serine and acidic residues were most frequently associated with fluoroquinolone resistance [2,6,7,19,20,21], the mechanistic underpinnings of this resistance did not become clear until the discovery of the water–metal ion bridge. A series of biochemical studies with purified gyrase and topoisomerase IV confirmed the biological relevance of the water–metal ion bridge, demonstrated that it serves as the primary conduit between fluoroquinolones and the bacterial type II topoisomerases, and established that mutations that disrupted bridge function were the fundamental cause of fluoroquinolone resistance [2,6,7,22,23,24,25].

Even though fluoroquinolones act on both gyrase and topoisomerase IV, their activity within bacterial cells is generally “unbalanced” in nature [4,6,10,26]. In all Gram-negative species examined to date, gyrase is the primary cellular target of fluoroquinolones, with topoisomerase IV contributing secondarily to drug efficacy [4,6,10,26]. A similar targeting pattern has been observed in most Gram-positive organisms, with the exceptions of *Staphylococcus aureus* and *Streptococcus pneumoniae*. In these two species, topoisomerase IV rather than gyrase functions as the primary lethal target of fluoroquinolones [4,6,10,26,27,28,29]. This unbalanced targeting has profound consequences for the evolution of drug resistance. In many cases, a single mutation in one type II enzyme confers sufficient resistance to permit survival during fluoroquinolone treatment and facilitate the acquisition of additional mutations, ultimately leading to highly resistant strains [4,6,26].

For many years, the success of fluoroquinolones as a broad-spectrum class of antibacterials discouraged further exploration of gyrase and topoisomerase IV as targets for additional antibacterial agents [6]. However, the increasing prevalence of fluoroquinolone resistance created an opportunity to develop new antibacterial classes that act on these validated targets while overcoming resistance mechanisms associated with the water–metal ion bridge [6,7].

## 3. Novel Gyrase/Topoisomerase IV-Targeted Antibacterials

Nearly 40 years after fluoroquinolones were introduced for clinical practice, two new classes of gyrase/topoisomerase IV-targeted antibacterials, the triazaacenaphthylenes and the spiropyrimidinetriones, were approved for use in humans earlier this year.

### 3.1. Gepotidacin

**Figure 2:**
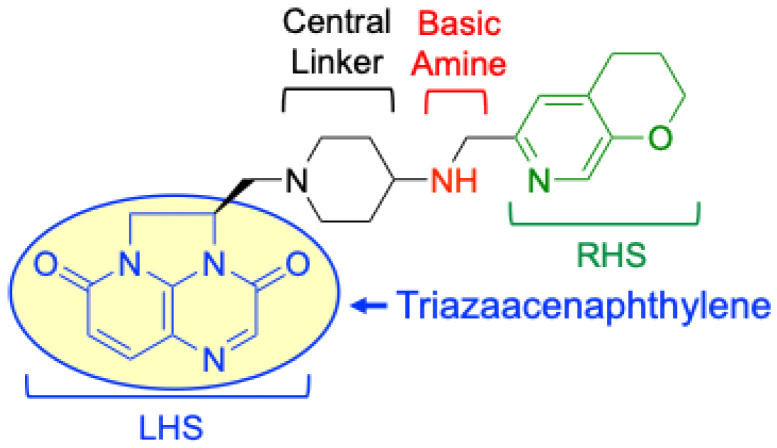
Structure of the triazaacenaphthylene gepotidacin with key pharmacophoric elements shown: left-hand side (LHS, blue) triazaacenaphthylene moiety (yellow) that interacts with two central base pairs of the DNA cleavage site, central linker (black), basic amine (red) that interacts with the aspartic acid (*E. coli* GyrA^D92^), and right-hand side (RHS, green) that binds in a hydrophobic pocket on the GyrA or ParC dimer interface.

Gepotidacin is a first-in-class triazaacenaphthylene (Figure 2) that targets gyrase and topoisomerase IV via a mechanism that is distinct from that of fluoroquinolones [2,6]. Whereas two fluoroquinolone molecules bind proximal to the two scissile DNA bonds (one molecule per scissile bond) within the enzyme–DNA complex [2,6,7], gepotidacin engages gyrase and topoisomerase IV as a single molecule [30,31,32]. Structural analyses of *Staphylococcus aureus* [31] and *E. coli* [32] gyrase reveal that the left-hand side (LHS, Figure 2) of gepotidacin is positioned midway between the two DNA scissile bonds and occupies a DNA pocket on the twofold axis of the cleavage complex. In contrast, the right-hand side (RHS) resides in a pocket on the twofold axis between the two GyrA subunits. A critical interaction with gepotidacin is mediated by an aspartic acid residue (predicted to be GyrA^D82^ and ParC^D86^ in *E. coli* gyrase and topoisomerase IV, respectively). This residue forms a hydrogen bond with the basic nitrogen of the gepotidacin skeleton [31,32]. This distinct binding mode underlies the potential of gepotidacin to overcome target-mediated fluoroquinolone resistance [30,31,32,33].

Gepotidacin is a potent inhibitor of gyrase and topoisomerase IV from diverse bacterial species and promotes DNA cleavage mediated by these enzymes [31,33,34,35,36]. However, in contrast to fluoroquinolones, which characteristically generate double-stranded DNA breaks [6], gepotidacin induces primarily single-stranded DNA breaks [31,33,36]. Although the mechanistic basis for the induction of single-stranded DNA breaks is not fully understood, it has been proposed that the interactions of the triazaacenaphthylene on the two-fold axis of the cleavage complex impose sufficient distortion after one DNA strand is cleaved to prevent cleavage of the second strand [31].

In the species that have been examined, gepotidacin retains activity against gyrase and topoisomerase IV that contain the most common fluoroquinolone-resistance mutations [33,36,37]. Similar results have been seen in cell lines that encode these mutations [33,37,38,39]. These findings predict that gepotidacin should maintain activity against many clinically important fluoroquinolone-resistant infections, which is consistent with its demonstrated efficacy against fluoroquinolone-resistant *Neisseria gonorrhoeae* isolates from participants in a recent phase III trial [40].

In addition to its ability to maintain activity against target-mediated fluoroquinolone resistance, a second advantage offered by gepotidacin is its well-balanced dual targeting of gyrase and topoisomerase IV (in at least some species). Studies with *E. coli*, *Klebsiella pneumoniae*, and *N. gonorrhoeae* indicate that gepotidacin can exert its bactericidal activity equally well through either enzyme and that reduced drug susceptibility requires the simultaneous accumulation of gepotidacin-specific mutations in both gyrase and topoisomerase IV [33,38,39]. Thus, target-mediated resistance to gepotidacin is expected to arise only when concurrent mutations occur in both enzymes. This well-balanced dual targeting predicts an extended clinical lifespan for gepotidacin, at least with respect to target-mediated resistance, compared with the fluoroquinolone class.

On 25 March 2025, gepotidacin was approved by the United States Food and Drug Administration (FDA) for the treatment of uncomplicated urinary tract infections in adult and adolescent females [41,42,43]. The drug was approved for the same indication in the United Kingdom by the Medicines and Healthcare products Regulatory Agency (MHRA) on 28 August 2025 [44]. Gepotidacin represents the first new class of antibacterials approved for the treatment of uncomplicated urinary tract infections in nearly three decades. Uncomplicated urinary tract infections account for more than eight million visits to healthcare providers, one million emergency room visits, and 100,000 hospitalizations annually in the United States [43,45,46,47]. Notably, approximately one-third of urinary tract infections caused by infection with *E. coli* (the most prevalent urinary tract infection pathogen) are resistant to fluoroquinolones [43,45,46,47]. Furthermore, it is estimated that more than 90% of all bacteria that cause urinary tract infections exhibit resistance to at least one commonly used class of antibacterials [43,45,46,47].

On 11 December 2025, gepotidacin was approved by the United States FDA for the treatment of uncomplicated urogenital gonorrhea [48]. Gonorrhea is a sexually transmitted disease that infects the mucosal epithelium of the genitals, rectum, and throat [49,50]. An estimated 82 million cases of gonorrhea occur worldwide each year [49,51]. The emergence and spread of antibacterial-resistant strains of *N. gonorrhoeae* has prompted the World Health Organization to issue a dire warning that the infection has the potential to join Hepatitis B, Herpes, Human Papillomavirus, and HIV/AIDS as an incurable sexually transmitted infection [52,53]. If untreated, gonorrhea can lead to severe complications, including pelvic inflammatory disease; infertility; and when disseminated, death [49,54]. Although ciprofloxacin was a first-line treatment for gonorrhea in the 1990s [55,56], it was removed from treatment guidelines in 2006 due to the widespread emergence of target-mediated resistance [57]. Currently, approximately 30–35% of clinical gonorrhea isolates in the United States are resistant to fluoroquinolones [58,59]. Resistance levels are considerably higher in the United Kingdom (~65%) and parts of Asia and Southeast Asia (85–95%) [58,59]. Gepotidacin is the first new class of antibacterials introduced for the treatment of gonorrhea in nearly 40 years, since the adoption of fluoroquinolones in the 1990s.

### 3.2. Zoliflodacin

**Figure 3:**
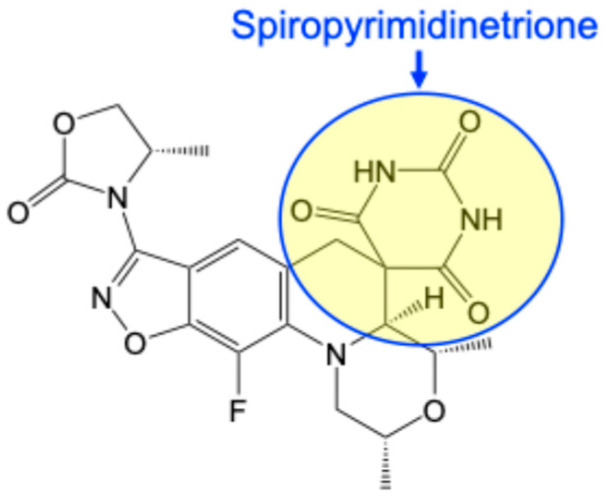
Structure of the spiropyrimidinetrione zoliflodacin. The drug class derives its name from the spiropyrimidinetrione moiety (yellow) that forms critical contacts with gyrase and topoisomerase IV and is essential for antibacterial activity.

The second novel class of antibacterials that target gyrase and topoisomerase IV is the spiropyrimidinetriones, with zoliflodacin (Figure 3) representing the first-in-class agent. The binding site for spiropyrimidinetriones in the gyrase/topoisomerase IV DNA cleavage complex overlaps that of fluoroquinolones. Similar to fluoroquinolones, spiropyrimidinetriones intercalate into the cleaved scissile bonds, with one molecule inserted into each opposing DNA strand [60,61]. This binding mode of zoliflodacin and related spiropyrimidinetriones underlies their ability to induce gyrase/topoisomerase IV-mediated double-stranded DNA breaks [62,63]. However, in contrast to the fluoroquinolones, which interact predominately with the GyrA side of the double helix, spiropyrimidinetriones engage the enzymes primarily on the opposite, or GyrB side [60,61,64]. The primary point of contact for spiropyrimidinetriones in the enzyme-DNA complex appears to be a highly conserved aspartic acid residue (equivalent to Asp426 in *E. coli* GyrB) [60,61,64,65].

The interaction between spiropyrimidinetriones and GyrB obviates the use of the fluoroquinolone water–metal ion bridge [60,61,62]. Consequently, zoliflodacin overcomes target-mediated fluoroquinolone resistance in cultured cells from a variety of species [65,66,67,68,69]. Although studies with purified gyrase and topoisomerase IV have only been reported for *N. gonorrhoeae* and *Mycobacterium tuberculosis*, zoliflodacin and related spiropyrimidinetriones retained their activity (or displayed higher activity) against fluoroquinolone-resistant enzymes [62,63,70,71,72].

At least in *N. gonorrhoeae*, zoliflodacin displays a low propensity to induce resistance [62,64]. However, as with the fluoroquinolones, the drug primarily targets gyrase in this species [62,63]. Whether this unbalanced cellular targeting will have clinical significance remains to be determined.

On 12 December 2025, zoliflodacin was approved by the United States FDA for the treatment of uncomplicated urogenital gonorrhea [73,74]. Remarkably, after nearly four decades without the introduction of a new antibacterial class for the treatment of gonorrhea, gepotidacin and zoliflodacin received FDA approved just one day apart [75]! Together, these two drugs provide a powerful and complementary addition to our arsenal of antibacterials for combating this critically important infection.

## 4. Conclusions

For more than four decades, gyrase and topoisomerase IV have been central to human health as the molecular targets of fluoroquinolones, one of the most widely prescribed classes of antibacterials. However, the clinical effectiveness of fluoroquinolones against several critical infections has been increasingly compromised by the emergence of target-mediated resistance. Historically, efforts to develop gyrase- and topoisomerase IV-directed antibacterials focused primarily on optimizing fluoroquinolone scaffolds to enhance potency and spectrum. More recently, this paradigm has shifted with the establishment of two new mechanistically distinct classes of gyrase/topoisomerase IV-targeted antibacterials, the triazaacenaphthylenes and the spiropyrimidinetriones. The first-in-class agents gepotidacin and zoliflodacin, respectively, were approved for human use earlier this year, marking a significant expansion of the therapeutic strategies available for targeting bacterial type II topoisomerases. These approvals reaffirm gyrase and topoisomerase IV as enduring antibacterial targets and validate the pursuit of mechanistically distinct strategies for overcoming fluoroquinolone resistance.

## Data Availability

No new data were created or analyzed in this study. Data sharing is not applicable to this article.
